# ﻿Phylogeny of *Macrobrachium* spp. (Decapoda, Pleocyemata) from Peru based on mitochondrial and nuclear data reveals a species complex comprising *M.digueti* (Bouvier, 1895) and *M.transandicum* Holthuis, 1950

**DOI:** 10.3897/zookeys.1224.130537

**Published:** 2025-01-20

**Authors:** Eliana Zelada-Mázmela, Lorenzo E. Reyes-Flores, Luis De Stefano-Beltrán

**Affiliations:** 1 Laboratory of Genetics, Physiology and Reproduction, National University of Santa, Av. Pacífico 508, Nuevo Chimbote, Ancash, Peru National University of Santa Nuevo Chimbote Peru; 2 Peruvian University Cayetano Heredia, Av. Honorio Delgado 430, San Martín de Porres, Lima, Peru Peruvian University Cayetano Heredia Lima Peru

**Keywords:** Genetic variability, *Macrobrachium*, molecular phylogenetics, *olfersii* species complex, Peruvian river prawns, systematics

## Abstract

Prawns of the genus *Macrobrachium* represent a highly diverse group of high commercial value worldwide. Eight *Macrobrachium* species have been reported from the Peruvian Pacific slope, but their phylogenetic relationships are still unknown. To investigate the systematics of *Macrobrachium* species from Peru, morphological identification and molecular data from nucleotide sequences of three genes were used: cytochrome *c* oxidase subunit I, 16S rRNA, and 28S rRNA. Based on morphological taxonomy, six species were successfully identified: *M.americanum*, *M.digueti*, *M.gallus*, *M.inca*, *M.panamense*, and *M.transandicum*. However, the phylogenetic inference results supported the species validity of only the first five species; all prawn individuals that were morphologically identified as *M.transandicum* were recovered within the *M.digueti* group, showing interspecific genetic distances near zero, suggesting that both species belong to the same species-level lineage, which may represent in the “*olfersii* species complex”. Our analyses also corroborated the genetic proximity of sibling species *M.inca*–*M.americanum* and *M.gallus*–*M.panamense*, and the monophyletic origin of *Macrobrachium* species from Peruvian populations. This study represents the first comprehensive phylogenetic analyses of *Macrobrachium* species from Peru, and contributes the first publicly available DNA sequences for *M.inca* and *M.gallus*, as well as the first sequences of *M.americanum*, *M.panamense*, *M.digueti*, and *M.transandicum* collected from Peruvian rivers.

## ﻿Introduction

Prawns of the genus *Macrobrachium* Spence Bate, 1868 are cosmopolitan species inhabiting freshwater and estuarine ecosystems (Bowles et al. 2000). This speciose crustacean genus currently encompasses 319 accepted species in the World Register of Marine Species (WoRMS Database 2024) database, many of them being of high commercial value worldwide (Makombu et al. 2019); however, several studies have suggested the existence of cryptic species causing taxonomic issues that are yet to be resolved (Liu et al. 2007). This crustacean group is also known for the presence of strong interspecific conservatism and intraspecific variation, which makes it taxonomically recalcitrant (Pileggi and Mantelatto 2010; Rossi and Mantelatto 2013), especially when systematics studies have been based mainly on comparison of external morphological traits (de Bruyn 2005; Nogueira et al. 2023). Arguably, the palaemonid prawn classification scheme given by Holthuis (1950, 1952) is the most widely used for the taxonomic classification of freshwater prawns from the Americas (Murphy and Austin 2002, 2003). However, the diagnostic characters determined by Holthuis have been critically debated due to their complex morphological variation (Murphy and Austin 2002, 2003).

More recently, this group has received special attention with studies mainly related to taxonomy and molecular systematics (Nogueira et al. 2023). Molecular phylogenetics has become a powerful tool and more studies are combining molecular and morphological data aiming to obtain a more robust insight into the classification of *Macrobrachium* species. Nuclear and mitochondrial DNA markers have been successfully used to solve taxonomic issues in highly diversified decapod groups (Murphy and Austin 2004; Siriwut et al. 2020 and references therein) including the description of cryptic species from *Macrobrachium* (Pileggi et al. 2014; Fuke and Imai 2018; Siriwut et al. 2020; Rossi et al. 2023). Despite the large diversity of *Macrobrachium* species and species complexes existing in populations from Latin America (García-Guerrero et al. 2013; Pileggi et al. 2014), to date most taxonomic studies on *Macrobrachium* have mainly focused on species from the Indo-Pacific region (where a higher number of *Macrobrachium* species occur), and only a few studies have used molecular and morphological data to analyze *Macrobrachium* populations from Brazil and Mexico (Pileggi and Mantelatto 2010; Rossi and Mantelatto 2013; Pileggi et al. 2014; García-Velazco et al. 2017; Rossi et al. 2023). A comprehensive systematic review of *Macrobrachium* by Anger (2013), concluded that *Macrobrachium* species from the Americas represent a separate group, including up to 57 species, of which three (*M.gallus* Holthuis, 1952, *M.inca* Holthuis, 1950, and *M.transandicum* Holthuis, 1950) are endemic to the western slopes of the Andes (Holthuis 1950; Anger 2013).

In Peru, eight *Macrobrachium* species (*M.gallus*, *M.inca*, *M.transandicum*, *M.americanum* Bate, 1868, *M.tenellum* (Smith, 1871), *M.digueti* (Bouvier, 1895), *M.hancocki* Holthuis, 1950, and *M.panamense* Rathbun, 1912) have been reported to occur on the Pacific slope (Amaya and Guerra 1976; Méndez 1981; Valencia and Campos 2007; Luque 2008; Hendrickx and Wicksten 2011, Campos 2014), but only the first three species are endemic to the Ecuadorian and Peruvian Pacific slope, while the latter five species also occur in Central America and Mexico (Valencia and Campos 2007; Hernández 2008; Mc Larney et al. 2010).

Currently, there is no established *Macrobrachium* prawn fishery in Peru, and as with other *Macrobrachium* species, as it is generally a complementary and artisanal activity associated with the rainy season. The organisms caught are consumed locally or marketed in places close to the fishing grounds. However, their widespread use means that fishing pressure is increasing and the availability of areas for natural production is decreasing, aggravated by pollution, which limits the potential of natural populations. (López-Uriostegui et al. 2013). Despite the economic and culinary importance of prawns, studies on Peruvian freshwater prawns are scarce and those that exist are mostly related to Cryphiops (Cryphiops) caementarius (Molina, 1782) from central and southern Peruvian rivers (Zacarías and Yépez 2008). Furthermore, official inland capture fishery statistics of different *Macrobrachium* prawns are registered using the generic term “river prawn” (PRODUCE 2023) with no species-specific records. This common practice can lead to serious conservation problems, highlighting the urgent need for more taxonomic and population studies of *Macrobrachium* species from Peru.

Prawns are key elements of the food chain from freshwater environments, playing a major role not only as omnivorous scavengers and detritus feeders, but also as prey for fish, birds, and reptiles. Furthermore, they are considered important ecosystem engineers (García-Guerrero et al. 2013). To the best of our knowledge, despite the ecological (*Macrobrachium* prawns are key to the functionality and health of aquatic ecosystem) and commercial value of freshwater prawns, to date no study has applied nuclear and mitochondrial DNA markers to study the phylogeny of *Macrobrachium* species from Peruvian populations. The present study aimed to analyze the phylogenetic relationships among six *Macrobrachium* species collected from Peruvian rivers of the Pacific slope using morphological and molecular data. Phylogenetic relationships were inferred based on partial sequences of two mitochondrial makers, namely cytochrome *c* oxidase subunit I and 16S ribosomal RNA (hereafter referred to as COI and 16S rRNA, respectively), and one nuclear gene fragment, namely 28S ribosomal RNA (hereafter referred to as 28S rRNA).

## ﻿Materials and methods

### ﻿Field sampling and morphological identification

**Figure 1.**
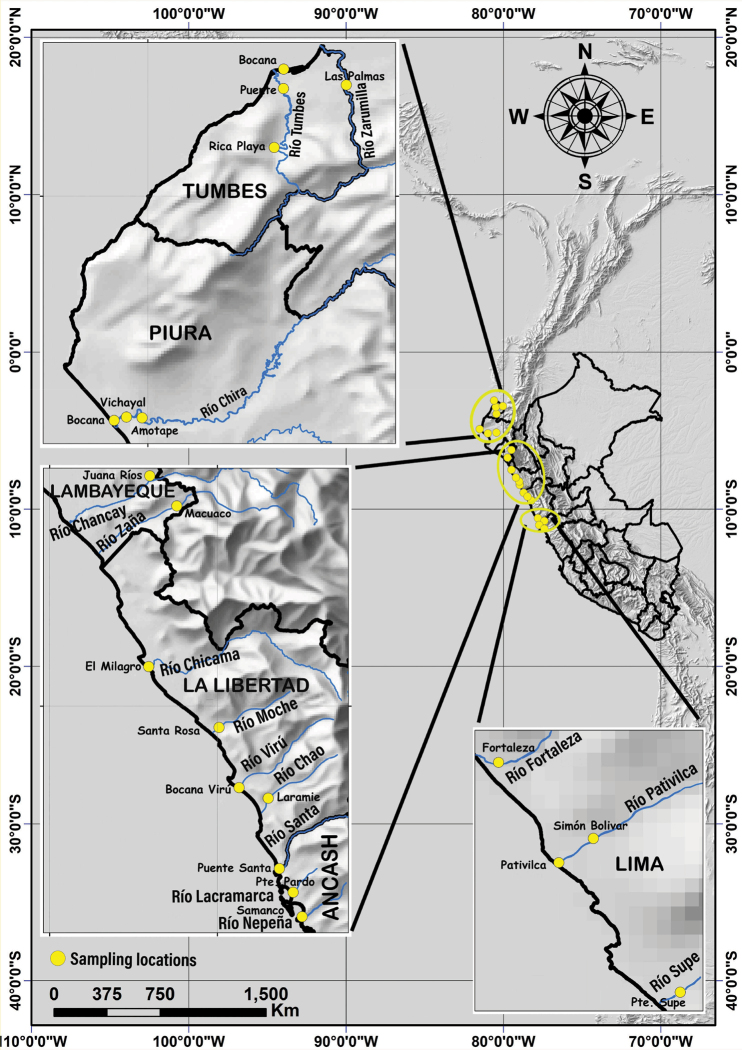
Geographic map of Peru showing sampling locations of *Macrobrachium* species (adapted from https://www.naturalearthdata.com/downloads/10m-raster-data/10m-shaded-relief/).

A total of 136 specimens belonging to the genus *Macrobrachium* were collected between December 2012 and February 2016 in rivers and estuaries from six Peruvian coastal regions including Tumbes, Piura, Lambayeque, La Libertad, Ancash, and Lima (Fig. 1; Suppl. material 2: table S1). The organisms were either bought from local fishermen or extracted using cast nets with a mesh size of 1 mm, landing nets, or by sieving seagrass beds and rocky bottoms of estuaries. Additionally, we also collected samples of the tropical river prawn *Palaemonhancocki* (Holthuis, 1950) to be used as an outgroup in phylogenetic analyses. Specimens were preserved in 96% ethanol, labeled by river name and collection date, and deposited in the voucher collection of the Laboratory of Genetics, Physiology, and Reproduction of the Universidad Nacional del Santa (**LGFyR-UNS**, Ancash, Peru). Morphological species identification was performed according to Méndez (1981) and Valencia and Campos (2007). The current accepted prawn scientific names and authorities were checked in WoRMS, which also includes the current revised taxonomy of freshwater species.

### ﻿DNA extraction, PCR amplification, and sequencing

Total genomic DNA was extracted from the muscle tissue of the pleopods, using the commercial GeneJET Genomic DNA Purification Kit (Thermo Fisher Scientific, Carlsbad, CA, USA). DNA quantification was calculated using an Epoch spectrophotometer (BioTek Instruments, Winooski, VT, USA). Extracted DNA quality was assessed by the 260/280 ratio and its integrity was observed by 1% agarose gel electrophoresis using GelRed Nucleic Acid Gel Stain as a DNA intercalator. Oligonucleotide sequences used for the polymerase chain reaction (PCR) amplification of partial fragments of COI, 16S rRNA, and 28S rRNA genes are shown in Table 1. All PCR amplifications were performed in a Veriti 96-Well Thermal Cycler (Applied Biosystems, Foster City, CA, USA) using Maximo *Taq* DNA Polymerase (GeneOn GmbH, Nurnberg, Germany) with the following master mix composition for COI y 16S rRNA: 1.14 μL of 25 mM MgCl_2_, 1.5 μL of 10X buffer, 0.75 μL of 2.5 mM dNTPs, 0.15 μL of each primer (50 μM), 0.15 μL of 5U μL^-1^ of *Taq* polymerase, 1 μL template DNA, and 10.16 μL of PCR Water (Invitrogen) to reach a total reaction volume of 15 μL. For 28S rRNA, the master mix composition was 1.3 μL of 25 mM MgCl_2_, 1.5 μL of 10X buffer, 0.75 μL of 2.5 mM dNTPs, 0.15 μL of each primer (50 μM), 0.15 μL of 5U μL^-1^ of *Taq* polymerase, 1 μL template DNA, and 10 μL of PCR Water (Invitrogen) to reach a total reaction volume of 15 μL. COI gene fragments were amplified with the following thermal cycler protocol: initial denaturation at 94 °C for 3 min, followed by 35 cycles of 94 °C for 45 s, 42 °C for 60 s, and 72 °C for 60 s, and a final extension step at 72 °C for 6 min. 16S rRNA gene fragments were amplified with the following thermal cycler protocol: initial denaturation at 94 °C for 3 min, followed by 30 cycles of 95 °C for 60 s, 40 °C for 60 s, and 72 °C for 60 s, and a final extension step at 72 °C for 10 min. 28S rRNA gene fragments were amplified with the following thermal cycler protocol: initial denaturation at 94 °C for 5 min, followed by 38 cycles of 94 °C for 30 s, 56 °C for 30 s, and 72 °C for 38 s, and a final extension step at 72 °C for 7 min. Successful PCR amplifications were verified in a 1% agarose electrophoresis using GelRed Nucleic Acid Gel Stain as a DNA intercalator. For DNA sequencing, all PCR products were purified using Exonuclease I and Shrimp Alkaline Phosphatase enzymes following Werle et al. (1994). Purified amplicons were Sanger sequenced bidirectionally by Macrogen Inc. (Rockville, MD, USA) in an ABI 3730Cl genetic analyzer (Applied Biosystems, Foster City, CA). For DNA electropherogram quality control, all sequences were manually checked and edited by removing ambiguous base callings, and COI sequences were checked for premature stop codons and frameshift indels that might indicate the presence of nuclear mitochondrial pseudogenes (NUMTs).

**Table 1.** Primer sequences used to amplify mitochondrial (COI and 16S rRNA) and nuclear (28S rRNA) genes.

Primer name	Direction	Sequence (5’ -- 3’)	Gene	Reference
Col6bF	Forward	ACAAATCATAAAGATATYGG	COI	Schubart and Heber (2006)
COH6R	Reverse	TADACTTCDGGRTGDCCAAARAAYCA		
1471	Forward	CCTGTTTANCAAAAACAT	16S rRNA	Munashinge (2010); Liu et al. (2007)
1472	Reverse	AGATAGAAACCAACCTGG		
28RDDF D2CFD45F	Forward	TACCGTGAGGGAAAGTTGAAA	28S rRNA	Suresh et al. (2012); Ndong et al. (2012)
28D2CRD45R	Reverse	AGACTCCTTGGTCCGTGTTT		

### ﻿DNA sequence and genetic distance analyses

A matrix containing multi-aligned sequences was constructed for each gene analyzed gene (COI, 16S rRNA, and 28S rRNA) using all the obtained sequences from *M.americanum*, *M.digueti*, *M.panamense*, and *M.transandicum*. In each matrix, we included sequences from three specimens of *M.gallus* and *M.inca* collected from each river of our field surveys, avoiding common or shared haplotypes. *Palaemonhancocki* was used as an outgroup. All DNA sequences obtained in this study have been deposited in GenBank/EMBL/DDBJ databases with accession numbers from OR941326–OR941602 (Suppl. material 2: table S2). All sequences were multi-aligned using the ClustalW algorithm as implemented in MEGA v. 7.0.21 (Kumar et al. 2016). Intraspecific and interspecific pairwise genetic distances were calculated using the Kimura 2-parameter model using MEGA v. 7.0.21. Basic sequence analysis statistics such as nucleotide composition, conserved sites, variable sites, parsimony informative sites, transitions and transversions rates, and amino acid composition were determined using MEGA v. 7.0.21, considering the start codon nucleotide position for the COI gene. The complete amino acid COI sequence from *M.rosenbergii* (De Man, 1879) (GenBank accession AY659990) was used to determine the correct start codon position in our partial COI fragment sequences.

### ﻿Phylogenetic analyses

Three different phylogenetic methods were performed for each of the three selected genes including maximum parsimony (MP), maximum likelihood (ML) and Bayesian inference (BI), implemented in PAUP v. 4.0 (Swofford 2002), RAxML v. 8.2.13 (Stamatakis 2014), and MrBayes v. 3.2.2 (Ronquist et al. 2011) respectively. For the construction of the MP phylogenetic tree, which treats gaps as a fifth state character, node reliability was evaluated using 1000 bootstrap replicates. The ML approach was performed with default parameters and employing the GTRGAMMA model of evolution, using 1000 bootstrap replicates to verify tree topology and clade support. The BI approach, which is a probabilistic model of multiple sequence alignments that accounts for insertion and deletion events in addition to substitution (Palero and Crandall 2009), was performed using two independent runs, each with four Markov chains under the Metropolis-Hasting algorithm (MCMC). To find the best-fit model of evolution we used jModelTest 2 (Darriba et al. 2012) under the Bayesian Information Criterion (BIC). The analyses were run for 1,000,000 generations with sampling every 100 generations, until reaching a standard deviation of less than 0.01. The first 25% of the sampled trees were discarded as burn-in. All phylogenetic trees were drawn using the Figtree v. 1.4.2 program (Rambaut 2014). Additionally, aiming to obtain further evolutionary insights that might not be resolved with single gene phylogenetic analysis, three different concatenated gene datasets (COI-16S rRNA, COI-28S rRNA, 16S rRNA-28S rRNA) were constructed using SeaView v. 4.5.4 (Gouy et al. 2010). jModelTest 2 (Darriba et al. 2012) under BIC was used to find the best-fit model of evolution of the concatenated gene datasets. MP, ML and BI analyses were performed using the same parameters described above for phylogenetic analysis of a single gene locus. Substitution saturations in single codon positions from each COI and the level of nucleotide substitution and genetic variability in the 16S rRNA and 28S rRNA genes were evaluated using an entropy-based index as implemented in DAMBE 6 (Xia 2017).

## ﻿Results

### ﻿Morphological and molecular species identification

Among the 136 collected specimens, a total of six *Macrobrachium* species (Suppl. material 2: table S2) were identified based on morphological analyses following the taxonomic key reported by Méndez (1981) and Valencia and Campos (2007) (Suppl. material 1: figs S1–S9; Suppl. material 2: table S3).

### ﻿Phylogenetic relationships of COI dataset

**Figure 2.**
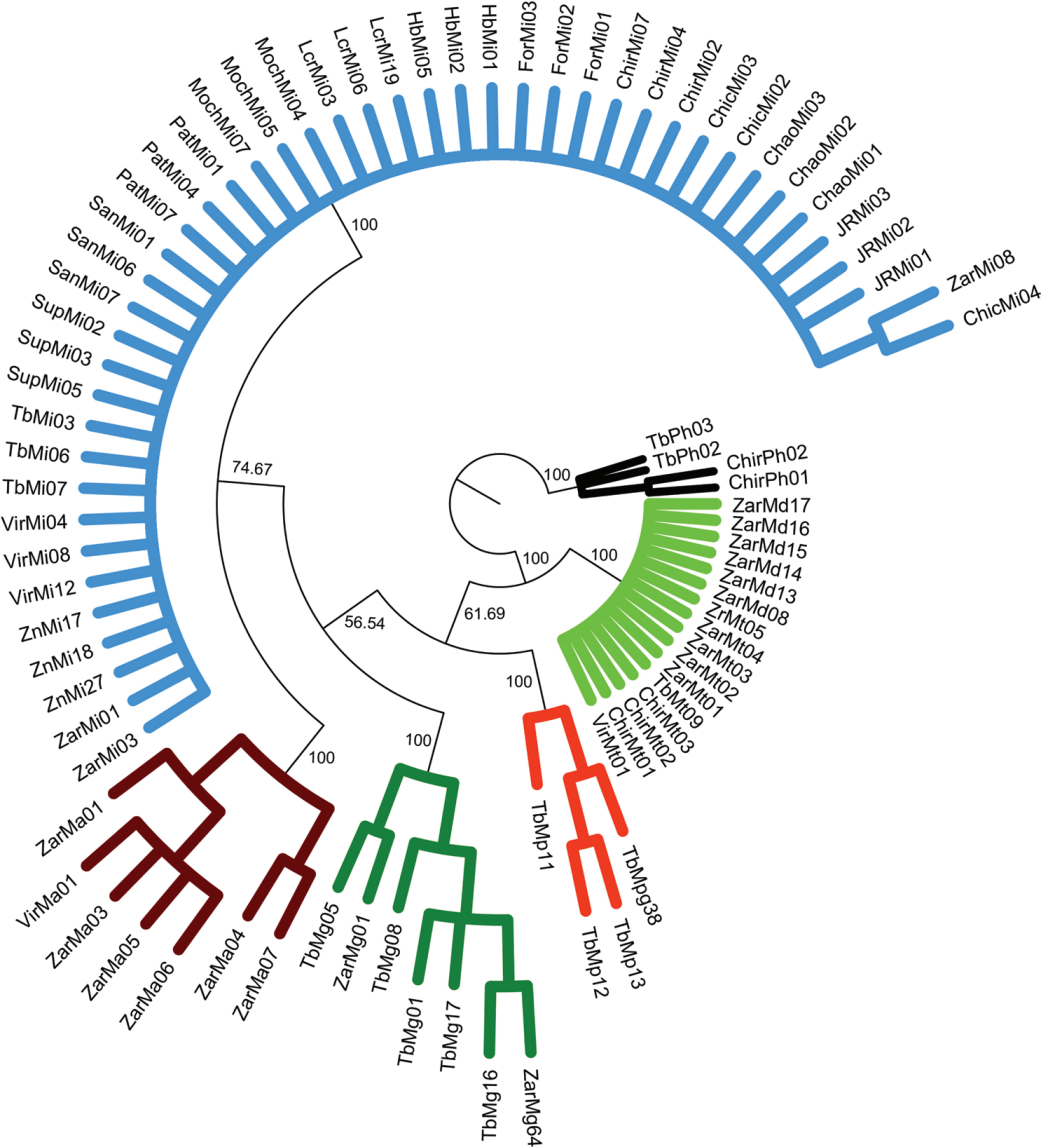
Phylogenetic tree based on maximum parsimony generated using 83 partial sequences of the mitochondrial COI gene from six *Macrobrachium* species collected in Peruvian rivers of the Pacific slope. Bootstrap values ≥ 50% are shown. *P.hancocki* was used as outgroup. GenBank accession numbers OR941326–OR941408. Abbreviations: Mt: *M.transandicum*; Mp: *M.panamense*; Mi: *M.inca*; Mg: *M.gallus*; Ma: *M.americanum*; Md: *M.digueti*, Ph: *P.hancocki*; Chir: Chira River; Tb: Tumbes River; Zr, Zar: Zarumilla River; Vir: Virú River; Pat: Pativilca River; JR: Juana Ríos River; Chao: Chao River; Lcr: Lacramarca River; Hb: Nepeña River; For: Fortaleza River; San: Santa River; Moch: Moche River; Chic: Chicama River; Sup: Supe River; Zn: Zaña River.

**Figure 3.**
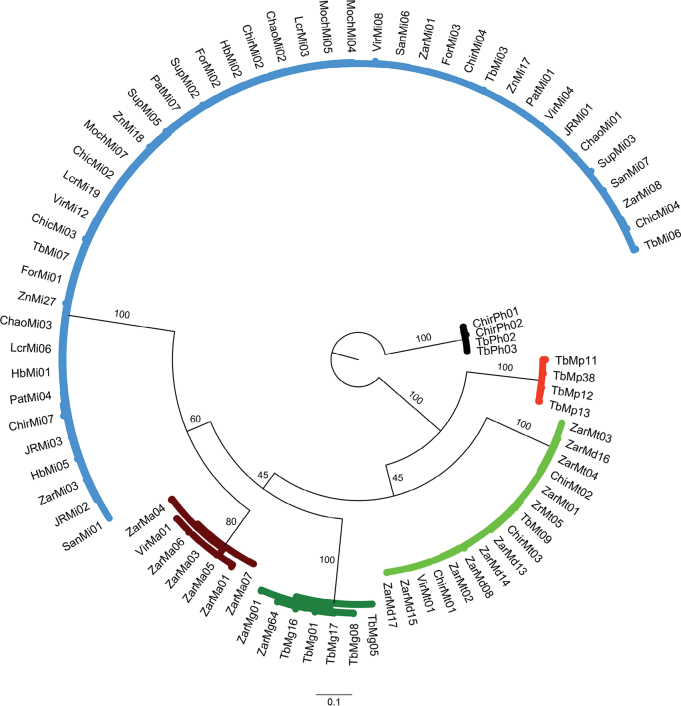
Phylogenetic tree based on maximum likelihood inference generated under the GTRGAMMA substitution model using 83 partial sequences of the mitochondrial COI gene from six *Macrobrachium* species collected in Peruvian rivers of the Pacific slope. Bootstrap values ≥ 50% are shown. *P.hancocki* was used as outgroup. GenBank accession numbers OR941326–OR941408. Abbreviations: Mt: *M.transandicum*; Mp: *M.panamense*; Mi: *M.inca*; Mg: *M.gallus*; Ma: *M.americanum*; Md: *M.digueti*; Ph: *P.hancocki*; Chir: Chira River; Tb: Tumbes River; Zr, Zar: Zarumilla River; Vir: Virú River; Pat: Pativilca River; JR: Juana Ríos River; Chao: Chao River; Lcr: Lacramarca River; Hb: Nepeña River; For: Fortaleza River; San: Santa River; Moch: Moche River; Chic: Chicama River; Sup: Supe River; Zn: Zaña River.

**Figure 4.**
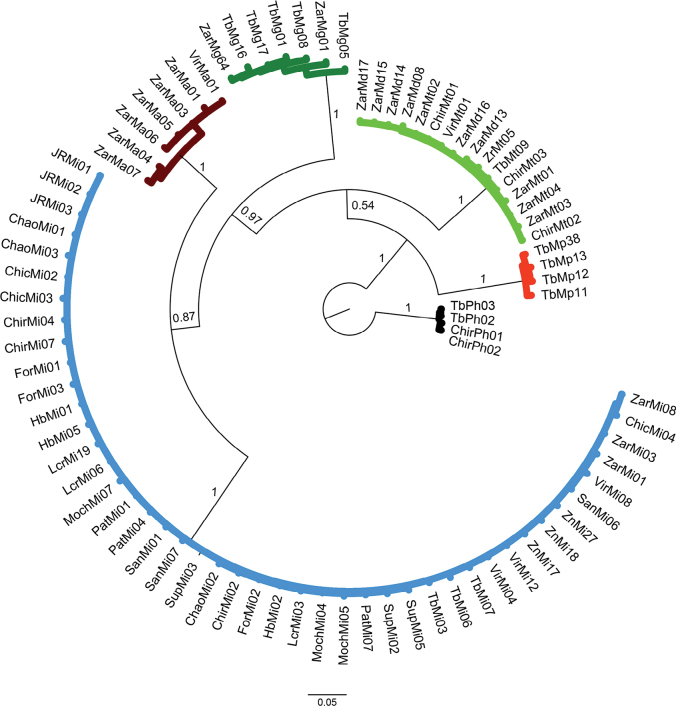
Phylogenetic tree based on Bayesian Inference approach generated under the GTR+I+G substitution model using 83 partial sequences of the mitochondrial COI gene from six *Macrobrachium* species collected in Peruvian rivers of the Pacific slope. Bootstrap values ≥ 50% are shown. *P.hancocki* was used as outgroup. GenBank accession numbers OR941326–OR941408. Abbreviations: Mt: *M.transandicum*; Mp: *M.panamense*; Mi: *M.inca*; Mg: *M.gallus*; Ma: *M.americanum*; Md: *M.digueti*; Ph: *P.hancocki*; Chir: Chira River; Tb: Tumbes River; Zr, Zar: Zarumilla River; Vir: Virú River; Pat: Pativilca River; JR: Juana Ríos River; Chao: Chao River; Lcr: Lacramarca River; Hb: Nepeña River; For: Fortaleza River; San: Santa River; Moch: Moche River; Chic: Chicama River; Sup: Supe River; Zn: Zaña River.

Overall, the results of phylogeny estimation approaches (MP, ML and BI) inferred with single and concatenated gene datasets showed similar topologies, branch lengths, and high bootstrap support and posterior probabilities. All approaches (MP, ML, and BI) (Figs 2–4, respectively) for the COI dataset (n = 83 sequences) showed that the six *Macrobrachium* species included in our analyses were recovered in five discrete clades: *M.inca* (Mi, n = 45), *M.gallus* (Mg, n = 7), *M.americanum* (Ma, n = 7), *M.panamense* (Mp, n = 4), and a single clade that grouped both *M.digueti* (Md, n = 6) and *M.transandicum* (Mt, n = 10). *Palaemonhancocki* (Ph, n = 4) was used as the outgroup. All substitution models used in our phylogenetic analyses are shown in Suppl. material 2: table S4. The MP, ML, and BI phylogenetic trees for COI sequences recovered *M.inca* and *M.americanum* in two sister clades with high bootstrap support (74.7, 60%) and posterior probabilities (87%), which is consistent with shared morphological characteristics between both species (short rostrum and similar shape of the second pair of pereiopods). Intraspecific genetic distance values of COI for the six *Macrobrachium* species analyzed in this study are shown in Table 2, ranging from 0.43% in *M.transandicum* to 1.78% in *M.americanum*. Interspecific genetic distances for COI (Table 3) ranged from 0.53% (between *M.transandicum* and *M.digueti*) to 23.9% (between *M.digueti* and *M.inca*). The short interspecific genetic distance found between *M.digueti* and *M.transandicum* (0.53%) caused the recovery of both species into a single clade in all phylogenetic trees (MP, ML and BI) with high statistical support (100% bootstrap value and posterior probabilities).

**Table 2.** Intraspecific genetic distances based on a partial fragment of the COI gene. Analyses were conducted using the Kimura 2-parameter model (K2P) with 1000 bootstrap replicates.

Species	Genetic distance (d)	Standard error (SE)
*M.inca*	0.007875916	0.001348432
*M.gallus*	0.016703893	0.002978309
*M.panamense*	0.009035682	0.002716602
*M.transandicum*	0.004338227	0.0015591
*M.digueti*	0.006141983	0.001761096
*M.americanum*	0.017789742	0.003291708
*P.hancocki*	0.003437498	0.001656147

**Table 3.** Interspecific genetic distances (below diagonal) based on a partial fragment of the COI gene. Analyses were conducted using the Kimura 2-parameter model (K2P) with 1000 bootstrap replicates. Standard error estimates are shown above the diagonal.

Species	*M.inca*	*M.americanum*	*M.digueti*	*M.panamense*	*M.transandicum*	*M.gallus*	*P.hancocki*
*M.inca*	–	0.017348	0.020433	0.021025	0.020447	0.019419	0.022040
*M.americanum*	0.179606	–	0.018468	0.017899	0.018470	0.018310	0.021414
*M.digueti*	0.238992	0.188721	–	0.019072	0.001463	0.017165	0.019316
*M.panamense*	0.228502	0.186465	0.203847	–	0.019046	0.021432	0.021106
*M.transandicum*	0.237390	0.188609	0.005268	0.202176	–	0.017119	0.019351
*M.gallus*	0.213766	0.198608	0.189913	0.236212	0.187403	–	0.021735
*P.hancocki*	0.262074	0.238108	0.211475	0.241439	0.210927	0.253760	–

### ﻿Phylogenetic relationships of 16S rRNA dataset

**Figure 5.**
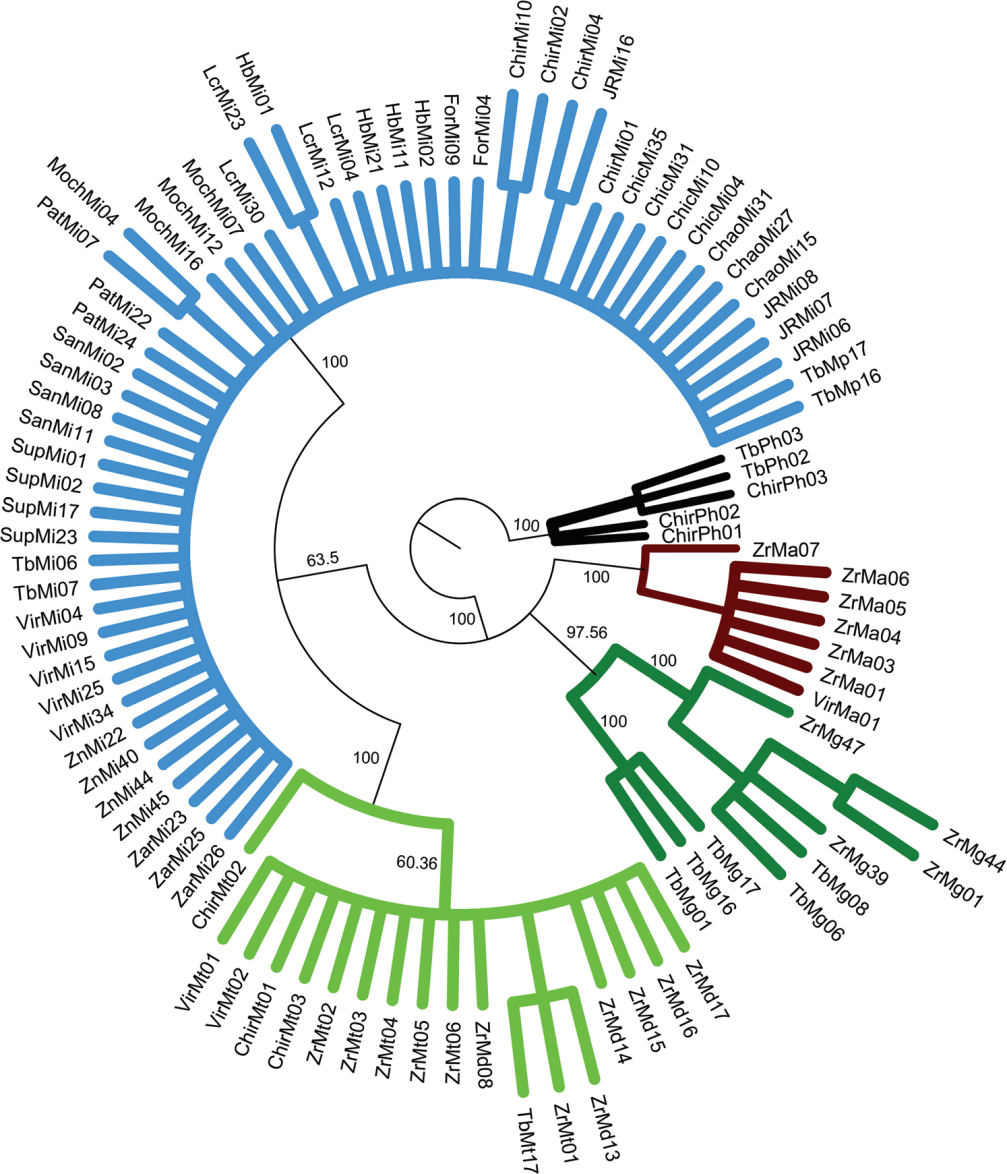
Phylogenetic tree based on Maximum Parsimony approach generated using 93 partial sequences of the mitochondrial 16S rRNA gene from six *Macrobrachium* species collected in Peruvian rivers of the Pacific slope. Bootstrap values ≥ 50% are shown. *P.hancocki* was used as outgroup. GenBank accession numbers OR941603–OR941697. Abbreviations: Mt: *M.transandicum*; Mp: *M.panamense*; Mi: *M.inca*; Mg: *M.gallus*; Ma: *M.americanum*; Md: *M.digueti*; Ph: *P.hancocki*; Chir: Chira River; Tb: Tumbes River; Zr, Zar: Zarumilla River; Vir: Virú River; Pat: Pativilca River; JR: Juana Ríos River; Chao: Chao River; Lcr: Lacramarca River; Hb: Nepeña River; For: Fortaleza River; San: Santa River; Moch: Moche River; Chic: Chicama River; Sup: Supe River; Zn: Zaña River.

**Figure 6.**
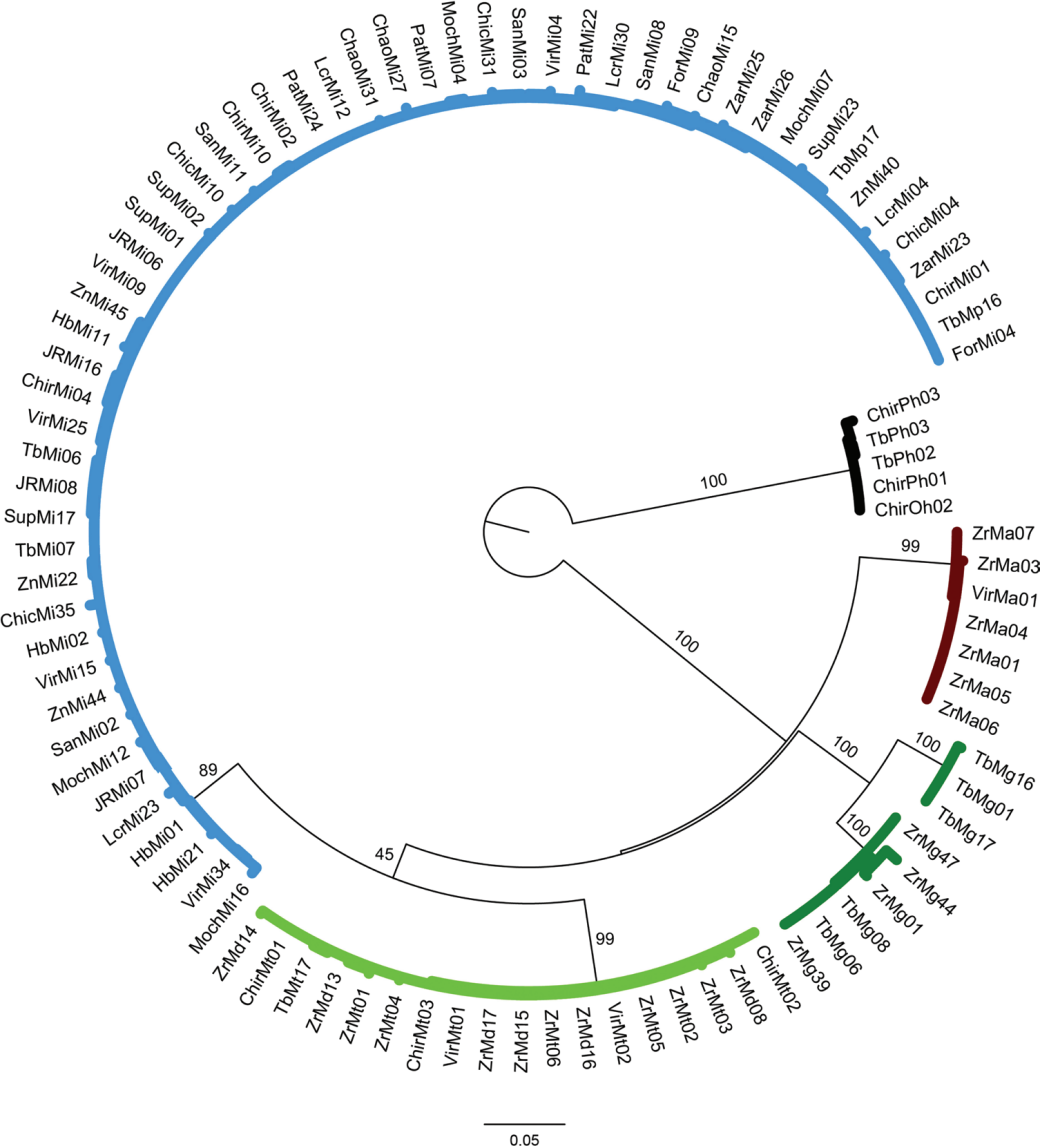
Phylogenetic tree based on maximum likelihood inference generated under the GTRGAMMA substitution model using 93 partial sequences of the mitochondrial 16S rRNA gene from six *Macrobrachium* species collected in Peruvian rivers of the Pacific slope. Bootstrap values ≥ 50% are shown. *P.hancocki* was used as outgroup. GenBank accession numbers OR941603–OR941697. Abbreviations: Mt: *M.transandicum*; Mp: *M.panamense*; Mi: *M.inca*; Mg: *M.gallus*; Ma: *M.americanum*; Md: *M.digueti*; Ph: *P.hancocki*; Chir: Chira River; Tb: Tumbes River; Zr, Zar: Zarumilla River; Vir: Virú River; Pat: Pativilca River; JR: Juana Ríos River; Chao: Chao River; Lcr: Lacramarca River; Hb: Nepeña River; For: Fortaleza River; San: Santa River; Moch: Moche River; Chic: Chicama River; Sup: Supe River; Zn: Zaña River.

**Figure 7.**
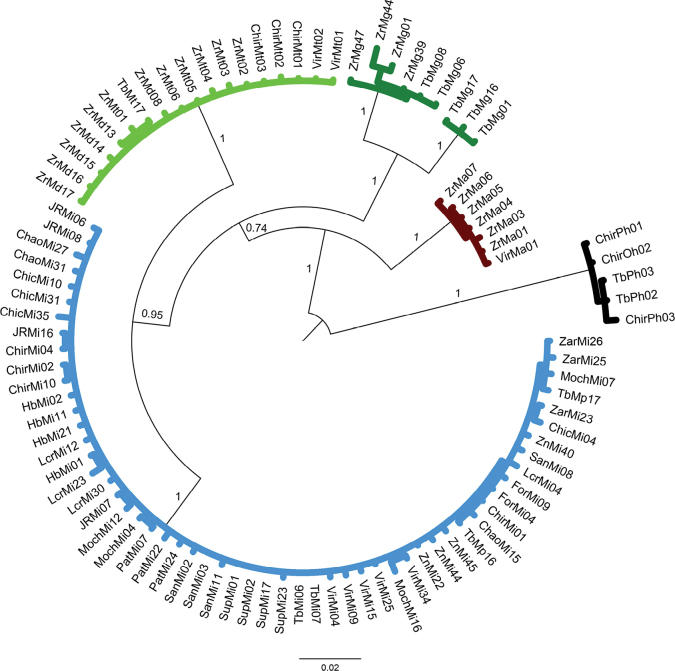
Phylogenetic tree based on Bayesian Inference approach generated under the K80+I+G substitution model using 93 partial sequences of the mitochondrial 16S rRNA gene from six *Macrobrachium* species collected in Peruvian rivers of the Pacific slope. Bootstrap values and posterior probabilities ≥ 50% are shown. *P.hancocki* was used as outgroup. GenBank accession numbers OR941603–OR941697. Abbreviations: Mt: *M.transandicum*; Mp: *M.panamense*; Mi: *M.inca*; Mg: *M.gallus*; Ma: *M.americanum*; Md: *M.digueti*; Ph: *P.hancocki*; Chir: Chira River; Tb: Tumbes River; Zr, Zar: Zarumilla River; Vir: Virú River; Pat: Pativilca River; JR: Juana Ríos River; Chao: Chao River; Lcr: Lacramarca River; Hb: Nepeña River; For: Fortaleza River; San: Santa River; Moch: Moche River; Chic: Chicama River; Sup: Supe River; Zn: Zaña River.

16S rRNA gene phylogenetic trees based in MP (Fig. 5), ML (Fig. 6), and BI (Fig. 7) showed similar topologies. In all trees, the monophyletic clades that recovered all *M.gallus* and *M.inca* sequences were placed in the basal and apical position of the tree, respectively. However, the phylogenetic trees recovered the two sequences representing *M.panamense* (TbMp16 GenBank accession OR941679 and TbMp17 GenBank accession OR941680) within the *M.inca* clade. Similar to the results obtained with the COI dataset, all sequences from *M.digueti* (Md, *n* = 6) and *M.transandicum* (Mt, *n* = 12) were recovered in a single clade with high bootstrap support (60% and 99%) and posterior probabilities (100%). The results of the intraspecific genetic distance values of the 16S rRNA gene dataset for the six *Macrobrachium* species analyzed in this study were found to be lower than those of the COI gene (Table 4) ranging from 0.11% in *M.americanum* to 0.72% in *M.gallus*. Interspecific genetic distances for 16S rRNA (Table 5) ranged from 0.19% (between *M.transandicum* and *M.digueti*) to 11.76% (between *M.gallus* and *M.digueti*). We should mention that COI and 16S rRNA gene sequences from *M.panamense* were obtained from different specimens and, in contrast to the COI genetic distance observed between *M.panamense* and *M.inca* (22.9%), the 16S rRNA distance observed for the same species pair was 0.53%, which was reflected in the recovery of both species within a single clade (Figs 2–4).

**Table 4.** Intraspecific genetic distances based on a partial fragment of the 16S rRNA gene. Analyses were conducted using the Kimura 2-parameter model (K2P) with 1000 bootstrap replicates.

Species	Genetic distance (d)	Standard error (SE)
*M.inca*	0.005080317	0.001149334
*M.americanum*	0.001143241	0.00077876
*M.digueti*	0.002001609	0.001112749
*M.panamense*	0.004003013	0.002731773
*M.transandicum*	0.001942077	0.000888224
*M.gallus*	0.007245067	0.002351216
*P.hancocki*	0.002803217	0.001658205

**Table 5.** Interspecific genetic distances (below diagonal) based on a partial fragment of the 16S rRNA gene. Analyses were conducted using the Kimura 2-parameter model (K2P) with 1000 bootstrap replicates. Standard error estimates are shown above the diagonal.

Species	*M.inca*	*M.americanum*	*M.digueti*	*M.panamense*	*M.transandicum*	*M.gallus*	*P.hancocki*
*M.inca*	–	0.014232	0.012861	0.001992	0.012809	0.014017	0.023743
*M.americanum*	0.098973	–	0.014303	0.014626	0.014328	0.013072	0.023497
*M.digueti*	0.077575	0.095831	–	0.013265	0.000785	0.015049	0.023423
*M.panamense*	0.005348	0.101574	0.079840	–	0.013213	0.014356	0.024126
*M.transandicum*	0.077179	0.096172	0.001891	0.079458	–	0.015013	0.023423
*M.gallus*	0.102915	0.092403	0.117623	0.104548	0.117217	–	0.024178
*P.hancocki*	0.209718	0.207972	0.216827	0.213408	0.216353	0.229778	–

### ﻿Phylogenetic relationships of 28S rRNA dataset

**Figure 8.**
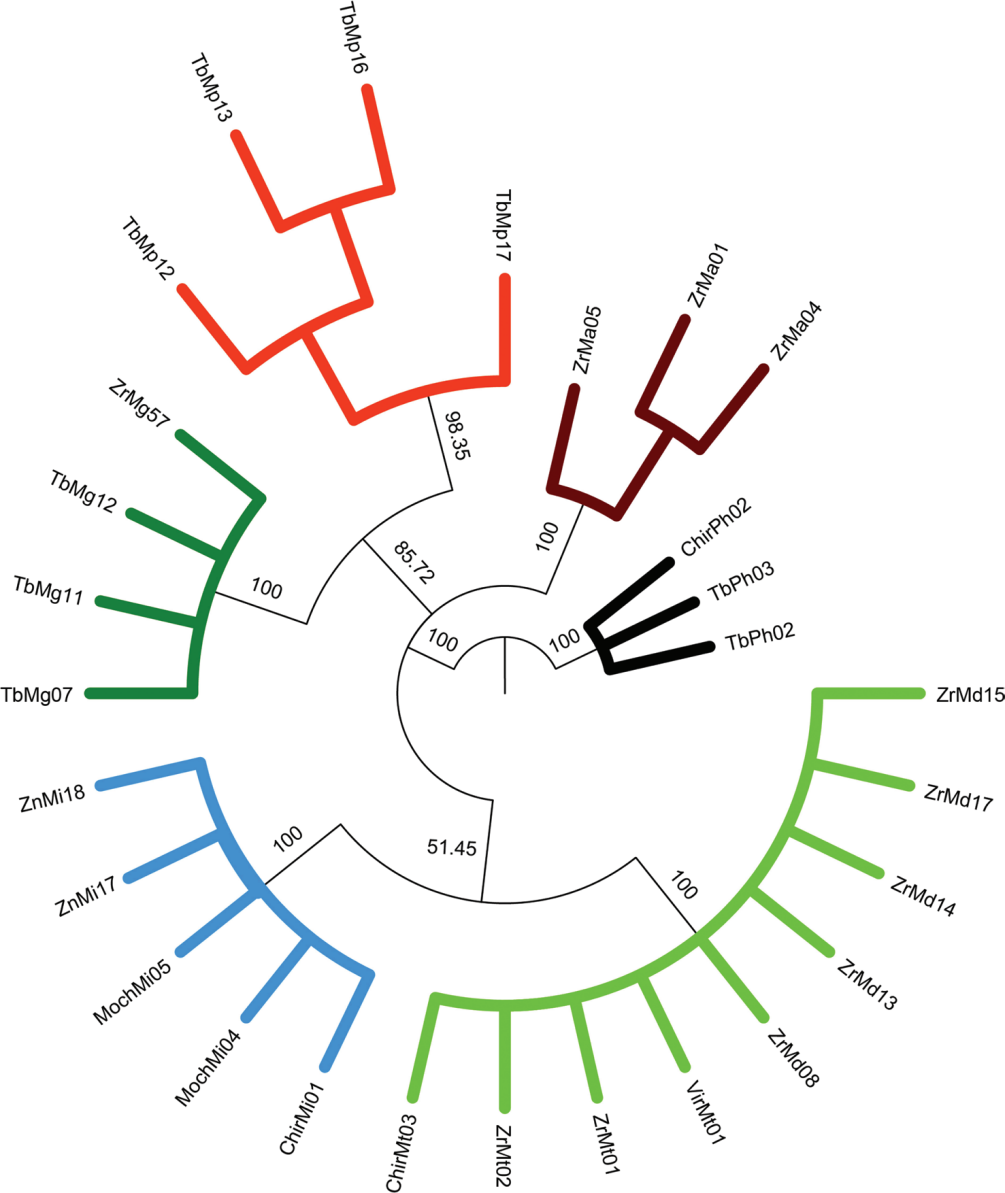
Phylogenetic tree based on Maximum Parsimony approach generated using 28 partial sequences of the nuclear 28S rRNA gene from six *Macrobrachium* species collected in Peruvian rivers of the Pacific slope. Bootstrap values ≥ 50% are shown. *P.hancocki* was used as outgroup. GenBank accession numbers OR941575–OR9411602. Abbreviations: Mt: *M.transandicum*; Mp: *M.panamense*; Mi: *M.inca*; Mg: *M.gallus*; Ma: *M.americanum*; Md: *M.digueti*; Ph: *P.hancocki*; Chir: Chira River; Tb: Tumbes River; Zr, Zar: Zarumilla River; Vir: Virú River; Pat: Pativilca River; JR: Juana Ríos River; Chao: Chao River; Lcr: Lacramarca River; Hb: Nepeña River; For: Fortaleza River; San: Santa River; Moch: Moche River; Chic: Chicama River; Sup: Supe River; Zn: Zaña River.

**Figure 9.**
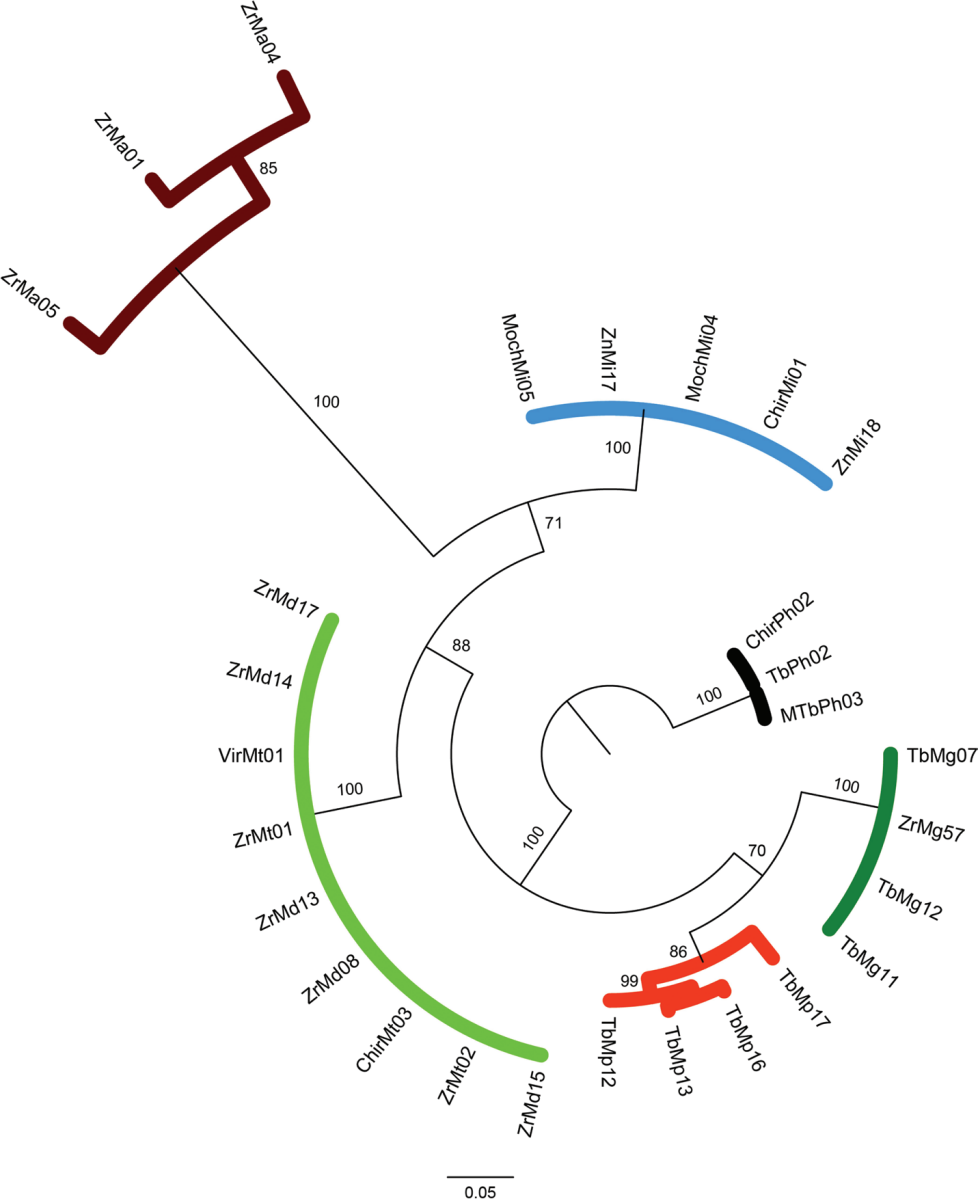
Phylogenetic tree based on maximum likelihood inference generated under the GTRGAMMA substitution model using 28 partial sequences of the nuclear 28S rRNA gene from six *Macrobrachium* species collected in Peruvian rivers of the Pacific slope. Bootstrap values ≥ 50% are shown. *P.hancocki* was used as outgroup. GenBank accession numbers OR941575–OR9411602. Abbreviations: Mt: *M.transandicum*; Mp: *M.panamense*; Mi: *M.inca*; Mg: *M.gallus*; Ma: *M.americanum*; Md: *M.digueti*; Ph: *P.hancocki*; Chir: Chira River; Tb: Tumbes River; Zr, Zar: Zarumilla River; Vir: Virú River; Pat: Pativilca River; JR: Juana Ríos River; Chao: Chao River; Lcr: Lacramarca River; Hb: Nepeña River; For: Fortaleza River; San: Santa River; Moch: Moche River; Chic: Chicama River; Sup: Supe River; Zn: Zaña River.

**Figure 10.**
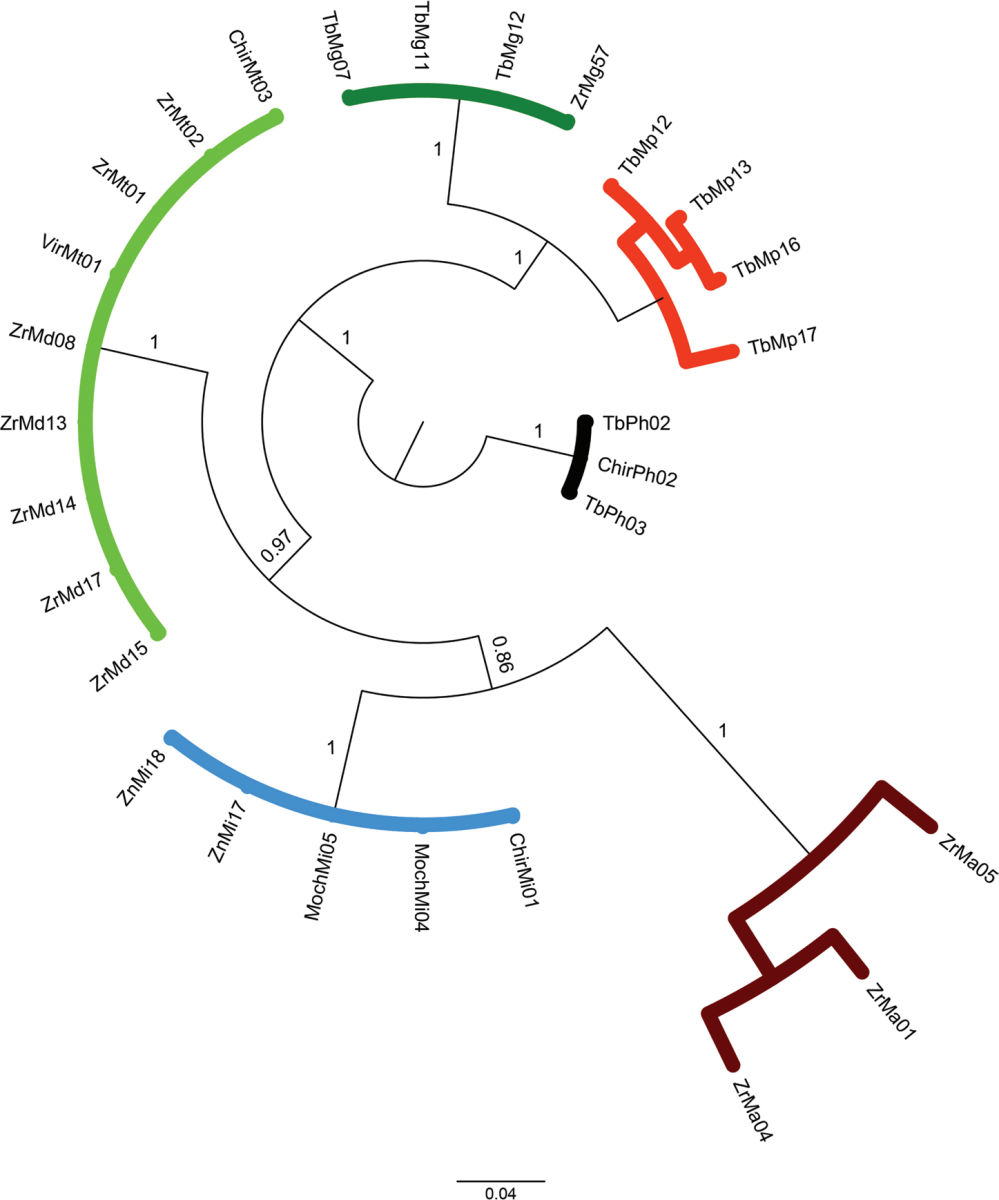
Phylogenetic tree based on Bayesian Inference approach generated under the GTR+I substitution model using 28 partial sequences of the nuclear 28S rRNA gene from six *Macrobrachium* species collected in Peruvian rivers of the Pacific slope. Bootstrap values and posterior probabilities ≥ 50% are shown. P*P.hancocki* was used as outgroup. GenBank accession numbers OR941575–OR9411602. Abbreviations: Mt: *M.transandicum*; Mp: *M.panamense*; Mi: *M.inca*; Mg: *M.gallus*; Ma: *M.americanum*; Md: *M.digueti*; Ph: *P.hancocki*; Chir: Chira River; Tb: Tumbes River; Zr, Zar: Zarumilla River; Vir: Virú River; Pat: Pativilca River; JR: Juana Ríos River; Chao: Chao River; Lcr: Lacramarca River; Hb: Nepeña River; For: Fortaleza River; San: Santa River; Moch: Moche River; Chic: Chicama River; Sup: Supe River; Zn: Zaña River.

The results from phylogenetic approaches (MP, Fig. 8; ML, Fig. 9 and BI, Fig. 10) for the 28S rRNA were highly similar, except for *M.digueti* (*n* = 5) and *M.transandicum* (*n* = 4) which were grouped in a single discrete clade with high statistical support (100% bootstrap value and posterior probability). All the other species (*M.americanum*, *M.gallus*, *M.inca*, and *M.panamense*) were recovered in unique clades with high bootstrap values (98–100%) and posterior probabilities (100%). The MP, ML, and BI phylogenetic tree results using the 28S rRNA dataset successfully recovered all *M.panamense* sequences in a single clade, including individuals TbMp16 (GenBank accession OR941594) and TbMp17 (GenBank accession OR941595) (see Figs 8–10), which under the 16S rRNA gene dataset were recovered within the *M.inca* group (see Figs 5–7).

The topology in the phylogenetic trees was also similar: *M.americanum* and *M.panamense* were recovered in two discrete clades with high bootstrap support (100 and 98%) and posterior probabilities (100%). The results also showed that the BI phylogenetic tree grouped *M.gallus* with *M.panamense* and *M.americanum* with *M.inca* in sister clades with high nodal support (100% and 86%, respectively). The two former species have a long rostrum while the two latter species share a similar long and robust shape of the second pair of pereiopods. As shown in Table 6, all the prawn species had no intraspecific genetic distance (0%) for the analyzed 28S rRNA fragment, except for *M.americanum* and *M.panamense* which displayed genetic distance values above zero (7.22% and 2.11% respectively). The calculated interspecific genetic distances for 28S rRNA among the *Macrobrachium* species analyzed herein resolved the taxonomic relationship between *M.panamense* and *M.inca*, showing a genetic distance of 12.3% (Table 7) with both species recovered in separate clades with high nodal support (MP: 98.3% and 100% respectively; ML: 96% and 100%, respectively; BI 100% for both species), supporting their status as different species and resolving the confusion as to their placement and classification obtained in the phylogenetic results from 16S rRNA. Our 28S rRNA data analysis results also revealed that there was no interspecific genetic distance gene between *M.digueti* and *M.transandicum* (0%, Table 7) confirming the phylogenetic grouping of these two species into a single discrete clade (MP, Fig. 8; ML, Fig. 9; BI, Fig. 10), which was also observed in the phylogenetic trees (MP, ML, and BI) from COI and the 16S rRNA gene datasets.

**Table 6.** Intraspecific genetic distances based on a partial fragment of the 28S rRNA. Analyses were conducted using the Kimura 2-parameter model (K2P) with 1000 bootstrap replicates.

Species	Genetic distance (d)	Standard error (SE)
*M.americanum*	0.072173333	0.008788746
*M.digueti*	0	0
*M.transandicum*	0	0
*M.gallus*	0	0
*M.inca*	0	0
*M.panamense*	0.021090406	0.004236636
*P.hancocki*	0	0

**Table 7.** Interspecific genetic distances (below diagonal) based on a partial fragment of the 28S rRNA. Analyses were conducted using the Kimura 2-parameter model (K2P) with 1000 bootstrap replicates. Standard error estimates are shown above the diagonal.

Species	*M.americanum*	*M.digueti*	*M.transandicum*	*M.gallus*	*M.inca*	*M.panamense*	*P.hancocki*
*M.americanum*	–	0.018854	0.018854	0.019530	0.019097	0.019233	0.020815
*M.digueti*	0.198629	–	0.000000	0.014078	0.013348	0.013639	0.016511
*M.transandicum*	0.198629	0.000000	–	0.014078	0.013348	0.013639	0.016511
*M.gallus*	0.213409	0.114759	0.114759	–	0.013662	0.010068	0.015985
*M.inca*	0.199487	0.112660	0.112660	0.112873	–	0.013677	0.016695
*M.panamense*	0.213907	0.117536	0.117536	0.077443	0.123234	–	0.014860
*P.hancocki*	0.224814	0.143690	0.143690	0.135800	0.155865	0.129517	–

### ﻿Phylogenetic relationships of concatenated datasets

Overall, the phylogenetic results obtained by using the three concatenated datasets COI-16S rRNA, COI-28S rRNA, and 16S rRNA-28S rRNA (Suppl. material 1: figs S10–S12) support the results obtained with the single locus phylogenetic analyses. However, it is worth noting that we did not test if the concatenated dataset COI-16S rRNA could have resolved the two *M.panamense* sequences (TbMp16 GenBank accession OR941679 - TbMp17 GenBank accession OR941680) that were grouped within the *M.inca* clade in the phylogenetic trees of 16S rRNA gene dataset (Figs 5–7), because we could not obtain good quality COI sequences from those two *M.panamense* individuals.

## ﻿Discussion

The diversity of freshwater crustacean decapods from South America is represented by seven families including Palaemonidae. In Peru, twelve Palaemonidae species occur naturally, of which eight belong to the genus *Macrobrachium* (Amaya and Guerra 1976) with only *M.inca* and *M.gallus* considered endemic to Peru (Zacarías and Yépez 2008), while *M.transandicum*, *M.digueti*, *M.panamense*, *M.americanum*, *M.tenellum*, and *M.hancocki* show a wider distribution range across the central Pacific (Holthuis 1950, 1952; Villalobos 1968; Valencia and Campos 2007; Hendrickx and Wicksten 2011; Campos 2014).

*Macrobrachium* presents low phenotypic variability, so species classification is usually very complicated, creating many taxonomic difficulties within the genus (Villalobos 1968; Pereira 1997; Murphy and Austin 2003, 2004; Pileggi and Mantelatto 2010). Despite this, the identification keys of Méndez (1981) and Valencia and Campos (2007) enabled morphological identification of the species.

During the morphological identification of *M.transandicum* we observed that both sexes displayed chelae of similar morphology (Suppl. material 1: figs S1–S4; Suppl. material 2: table S3). On the other hand, male individuals of *M.digueti* are characterized by having second pereiopods with unequal chelae (see Suppl. material 1: fig. S5) (Méndez 1981; Valencia and Campos 2007), which was a pivotal character for the successful morphological discrimination between both species, although the rostral formula was different from that reported by Valencia and Campos (2007) with 9–11 teeth versus 14–16/2–4. We also note that the rostrum of *M.transandicum* has more pronounced teeth than that of *M.digueti.* It has also been observed that chelae in *M.transandicum* are similar to those of the female *M.digueti* morphotype *michoacanus* Nates & Villalobos, 1990 reported by García-Velazco (2014). However, male and female individuals of *M.transandicum* identified in our study were found to have chelae of similar morphology between individuals of both sexes (Suppl. material 1: figs S1–S4). The issues found during our morphological identification are consistent with Pileggi and Mantelatto (2010) who suggested that the morphological characters frequently used in the identification of *Macrobrachium* species (rostral shape, rostral size, rostral teeth, telson spines, telson shape, morphology of the second pair of pereiopods) are not sufficient to resolve the taxonomic issues found in *Macrobrachium* species. Besides, those characters vary along the organism´s life span and are not common in both sexes.

To date, there is a lack of molecular studies of *Macrobrachium* species from Peru and most population study efforts have been focused on a single prawn species: C. (C.) caementarius, whose populations have been monitored periodically since 1996 by the Peruvian Marine Research Institute (IMARPE). Based on the reproductive periods determined for C. (C.) caementarius, a closed fishing season for all freshwater prawn species was established (Zacarías and Yépez 2008), including C. (C.) caementarius and *Macrobrachium* spp. (RM-312-2006-PRODUCE). In light of this, there is now an urgent need to conduct studies focused on the different *Macrobrachium* species that inhabit Peruvian ecosystems, including molecular data that can enable us to determine species delimitations and their phylogenetic relationships. An advantage of DNA sequence data is the higher taxonomic resolution over traditional systematics based on morphological characters alone (Murphy and Austin 2003, 2004; Murphy et al. 2004).

The present study represents the first effort to apply molecular data to analyze six of the eight different *Macrobrachium* species reported for Peru (Amaya and Guerra 1976), as well as contributing the first available sequences for *M.inca* and *M.gallus*, and the first sequences of *M.panamense*, *M.americanum*, *M.digueti*, and *M.transandicum* obtained from Peruvian rivers. Our genetic analyses using intra and interspecific distances and the recovered phylogenetic tree topologies based on single locus datasets corroborated the taxonomic category of species in five of the six analyzed species: *M.americanum*, *M.inca*, *M.gallus*, *M.panamense*, and *M.digueti*. On the other hand, *M.transandicum* showed very low genetic distances with *M.digueti* ranging from 0% for 28S rRNA (Table 7), 0.19% for 16S rRNA (Table 5), and 0.53% for COI (Table 3), reflected in the recovery of both species in a single clade in all phylogenetic trees obtained in this study (Figs 2–10, Suppl. material 1: figs S10–S12). Our results based on the analyses of three different molecular markers also suggest that *M.digueti* (Bouvier, 1895) and *M.transandicum* (Holthuis 1950) should be considered as a single species, with nomenclatural priority given to *M.digueti*. Similarly, Murphy and Austin (2004) using partial sequences of the mitochondrial 16S rRNA gene revealed that three different *Macrobrachium* species with considerable morphological variation were in fact only one species: *M.australiense* Holthuis, 1950. The authors reported genetic variation ranging from 0.2–1.6%, which is within the range of genetic distance detected in this study between *M.digueti* and *M.transandicum.* We also generated molecular operational taxonomic units (MOTUs) (Ramirez et al. 2023) and obtained the same results as those obtained with the sequences of the three genes in the study, i.e., *M.digueti* and *M.transandicum* form the same molecular operational unit (data not shown).

The geographic distribution of *M.transandicum* is not fully known and it has been reported that this species occurs only in three rivers in Colombia and one river in Peru (De Grave 2013). Previous studies have identified different morphotypes for *M.digueti*. For example, García-Velazco (2014) using the mitochondrial 16S rRNA gene reported a second morphotype of *M.digueti*, namely *M.michoacanus*, which was previously described as a different species habiting the Mexican Pacific slope. In the same study, a female holotype had a similar morphological appearance to *M.transandicum.* In the present work, we can rule out a misidentification of *M.transandicum* by a female of *M.digueti* because we were able to clearly identify individuals of both sexes in *M.transandicum* by the position of the gonopores (Suppl. material 1: figs S1–S4). Rossi and Mantelatto (2013) using sequences of the nuclear gene histone H3 recovered *M.digueti*, *M.olfersii* (Wiegmann, 1836), and *M.faustinum* (de Saussury, 1857) in a single clade, suggesting the existence of an “*olfersii* complex” encompassing several subspecies. We propose that *M.transandicum* should be also included in the *olfersii* complex.

### ﻿Phylogenetic relationships

The main objective of a molecular phylogenetics analysis is to infer the evolutionary history of a group of organisms and to output the results in a hierarchy branching diagram or phylogenetic tree (Palero and Crandall 2009). We chose the mitochondrial COI and 16S rRNA gene markers due to their high mutation rate (Rossi and Mantelatto 2013), and the nuclear 28S rRNA gene because it has been proven to be effective in previous studies of crustacean phylogenetics (Chen et al. 2009). The genetic distances among the different *Macrobrachium* species analyzed in our study (Tables 3, 5, 7) showed different evolutionary rates for each molecular marker, with COI being the best candidate for species discrimination and phylogenetic inferences of Peruvian *Macrobrachium* populations due to the relatively higher interspecific genetic distances observed in our results. This result agrees with previously related works. For example, Toon et al. (2009) reported that COI is highly variable among decapod species suggesting that it can be useful in resolving low-level taxonomy issues. In another work by Zhang et al. (2009), the authors used COI sequences to validate the status of species in *M.rosenbergii*, *M.nipponense* (De Haan, 1849), and *M.qilianensis* [unknown species according to WoRMS Database (2024), reporting high levels of interspecific genetic distances ranging from 19.87% to 23.84%. A more recent study by Siriwut et al. (2020) employed three molecular markers (COI, 16S rRNA, and 18S rRNA) for the phylogeny of *Macrobrachium* species from Thailand obtaining higher interspecific genetic distances with COI ranging from 9.8% to 23.3%. In the same study, the authors reported three new *Macrobrachium* species and remarked that the COI barcoding region provides the fine resolution required for the genus *Macrobrachium*.

The interspecific morphological conservation observed during the morphological identification of *Macrobrachium* is contrasted by the levels of genetic distances among species (Pileggi and Mantelatto 2010). Our phylogenetic analysis results based on the 16S rRNA gene showed a maximum interspecific genetic distance of 11.76% between *M.digueti* and *M.gallus* and a minimum of 0.1% between *M.digueti* and *M.transandicum* recovering the two latter species in a single clade. Similarly, *M.inca* and *M.panamense* were recovered in a single clade showing a low genetic distance of 0.5%. Thus, we can conclude that except for the case of *M.inca* and *M.panamense*, the 16S rRNA gene has enough resolution power and can be applied in phylogenetic studies of *Macrobrachium* species. Our results are consistent with previous crustacean phylogenetic studies based on the16S rRNA gene (Murphy and Austin 2004, 2005; Chan et al. 2008; Pileggi and Mantelatto 2010), which despite high evolutionary conservation, found interspecific divergence rates from 3.5% in decapods (Schubart 2009).

The addition of nuclear ribosomal genes for phylogeny studies of decapods has proven to be useful for different reasons including a lower evolutionary rate (Chu et al. 2009). Furthermore, previous phylogenetic studies of decapods including *Macrobrachium* based on both 28S rRNA and 16S rRNA gene markers detected some advantages of the former over the latter. Those advantages include a longer sequence length, a higher number of variable and parsimony informative sites, higher GC content, and a transition/transversion (TA/TV) rate ratio bias in favor of transitions over transversions (Crandall et al. 1999; Jarman et al. 2000; Porter et al. 2005; Chen et al. 2009). The results of the present study partially support previous findings showing that 28S rRNA sequences were 27% and 8% longer than those of the 16S rRNA and COI genes respectively, with higher GC content. However, TA/TV rate ratio was 0.99, biasing in favor of transversions over transitions. Increasing the sequence length also increases the number of informative sites, which in turn enhances the phylogenetic tree resolution (Chen et al. 2009). Furthermore, the inclusion of data from independent nuclear markers such as the 28S rRNA gene increases the possibility of recovering true phylogeny (Toon et al. 2009; Garrick et al. 2010). For example, the phylogenetic trees (MP, ML and BI) based on the 16S rRNA gene obtained in this study displayed misleading results of the true phylogenetic relationships between *M.panamense* and *M.inca*, recovering the only two *M.panamense* sequences (TbMp16 GenBank accession: OR941679 and TbMp17 GenBank accession: OR941680) within the *M.inca* clade (Figs 5–7). On the other hand, our phylogenetic analyses based on the 28S rRNA gene successfully resolved the phylogeny of *M.panamense* and *M.inca* recovering both species in separate discrete clades with high nodal support (98.3% to 100%) and posterior probabilities (100%) (Figs 8–10). We also note that the tree produced by ML and BI with 28S rRNA data (Figs 9, 10) recovered species of similar morphological characters in sister clades: *M.inca*–*M.americanum* (rostrum of medium size and second pair of pereiopods with unequal size), and *M.gallus*–*M.panamense* (long rostrum and thin and slender second pair of pereiopods); while the MP tree (Fig. 7) grouped only *M.gallus* and *M.panamense* in sister clades. These results support the hypothesis that *M.inca* and *M.gallus* are closely related to *M.americanum* and *M.panamense*, respectively. However, this hypothesis was not supported by the other two genes used in our phylogenetic inferences. We should expect a pattern of lower genetic distances between each pair of the closely related species than to the other *Macrobrachium* species considered in our analyses. Phylogenetic tree results based on partial COI gene fragments recovered this pattern only between *M.americanum* and *M.inca* but not between *M.gallus* and *M.panamense* (Figs 2–4).

Robust phylogenetic inference is achieved by using good datasets that usually depend on many sequences of long lengths. In this regard, the use of concatenated gene datasets represents a potentially powerful approach. However, this method should be used only with genes that show consistent evolutionary patterns (Palero and Crandall 2009). Our concatenated phylogenetic analysis results confirmed the results obtained with single locus datasets, determining the status of species in *M.panamense*, which was included within the *M.inca* clade in the results obtained by using the 16s rRNA gene dataset. Based on the recovered topology under both phylogenetic approaches (MP, ML and BI) using single and concatenated datasets (Figs 2–10), our results corroborated the monophyletic origin of *Macrobrachium* species from Peruvian populations of the Pacific slope. Similar results were reported for *Macrobrachium* species from Mexico (Acuña et al. 2013) and America (Pileggi and Mantelatto 2010). Contrastingly, previous phylogenetic studies reported the polyphyletic structure of *Macrobrachium* species from Australia and East/Southeast Asia using 16S rRNA (Murphy et al. 2004; Murphy and Austin 2005) and COI (Liu et al. 2007), respectively. Anger (2013) concluded that regardless of whether monophyly or paraphyly is assumed, all Paleo- and Neotropical *Macrobrachium* species originate from the same ancestor, and further species diversification resulted as part of the evolutionary process.

## ﻿Conclusions

Herein, we were able to identify and successfully recover phylogenetic relationships of six out of the eight *Macrobrachium* species reported for the Peruvian Pacific slope: *M.inca*, *M.gallus*, *M.transandicum*, *M.digueti*, *M.panamense*, and *M.americanum*. Two species, *M.tenellum* and *M.hancocki*, were not found in our field surveys and therefore not included in our study]. Based on our molecular analyses of partial fragments of COI, 16S rRNA, and 28S rRNA genes, the validity of five of these six species is supported; all our phylogenetic analyses recovered prawns morphologically identified as *M.transandicum* within the same clade as *M.digueti*, showing interspecific genetic distances near zero, and suggesting that both species belong to the same species-level lineage. Therefore, we propose that *M.transandicum* should be included in the *olfersii* complex.

Among the three molecular markers used in this study, we found that COI followed by 28S rRNA demonstrated strong resolving power for species identification and phylogenetic inferences of Peruvian *Macrobrachium* species. The 28S rRNA gene was also useful in resolving the taxonomic status of *M.panamense*. The hypothesis that *M.inca* and *M.gallus* are related to *M.americanum* and *M.panamense* respectively, was supported only by the BI phylogenetic tree based on 28S rRNA, whose topology recovered *M.inca* and *M.americanum* (rostrum of medium size and second pair of pereiopods with unequal size) and *M.gallus*–*M.panamense* (long rostrum and thin and slender second pair of pereiopods) in sister clades; while the COI trees recovered only the clade, *M.inca* and *M.americanum*. Finally, the phylogenetic approaches used in this study (MP, ML, and BI) recovered similar topologies for all the analyzed genes (COI, 16S rRNA, 28S rRNA), supporting the monophyletic origin of Peruvian *Macrobrachium* species.

## ﻿Additional information

### Author ORCIDs

Eliana Zelada-Mázmela https://orcid.org/0000-0002-5813-0954

Lorenzo E. Reyes-Flores https://orcid.org/0000-0002-1993-8010

Luis De Stefano-Beltrán https://orcid.org/0000-0002-5432-2916

### Data availability

All of the data that support the findings of this study are available in the main text or Supplementary Information.

## Supplementary materials

Supplementary material 1 Morphological identification of the six *Macrobrachium* species and Phylogenetic trees based on Bayesian inference Data type 7z Explanation note **fig. S1.***Macrobrachiumtransandicum* Holthuis, 1950. **A** Female (collected from Zarumilla River) **B** Rostrum in close-up view. **fig. S2.***Macrobrachiumtransandicum* Holthuis, 1950. Female (collected from Zarumilla River). **A** Major chela **B** Minor chela **C** Gonopore in coxae of the third pair of periopods. Note the width of the area around the fifth pair of periopods. **fig. S3.***Macrobrachiumtransandicum* Holthuis, 1950. **A** Male (collected from Tumbes River) **B** Rostrum in close-up view **C** Major chela. **fig. S4.***Macrobrachiumtransandicum* Holthuis, 1950. Male (collected from Tumbes River). **A** Minor chela **B** Gonopore, coxa fifth pair of periopods. Note the width of the area. **fig. S5.***Macrobrachiumdigueti* (Bouvier, 1895). **A** Male (collected from Zarumilla River). **B** Rostrum in close-up view **C** Major chela. **fig. S6.***Macrobrachiumpanamense* Rathbun, 1912. **A** Male (collected from Tumbes River) **B** Telson in close-up view **C** Rostrum close-up view. **fig. S7.***Macrobrachiumgallus* Holthuis, 1950. **A** Male (collected from Zarumilla River) **B** close-up view **C** Telson in close-up view. **fig. S8.***Macrobrachiumamericanum* Spence Bate, 1950. **A** Male (collected from Zarumilla River) **B** Telson in close-up view. **fig. S9.***Macrobrachiuminca* Holthuis, 1950. **A** Male (collected from Chicama River) **B** Rostrum in close-up view **C** Rostrum close-up view of a female (collected from Santa River) **D** Telson in close-up view. **fig. S10.** Phylogenetic tree based on Bayesian inference approach generated under the GTR+I+G substitution model using concatenated dataset of the mitochondrial COI-16S rRNA genes from six *Macrobrachium* species collected in Peruvian rivers of the Pacific. Bootstrap values and posterior probabilities ≥ 50% are shown. *P.hancocki* was used as outgroup. Abbreviations: Mt: *M.transandicum*; Mp: *M.panamense*; Mi: *M.inca*; Mg: *M.gallus*; Ma: *M.americanum*; Md: *M.digueti*; Ph: *P.hancocki*. **fig. S11.** Phylogenetic tree based on Bayesian inference approach generated under the GTR+I+G substitution model using concatenated dataset of COI-28S rRNA genes from six *Macrobrachium* species collected in Peruvian rivers of the Pacific. Bootstrap values and posterior probabilities ≥ 50% are shown. *P.hancocki* was used as outgroup. Abbreviations: Mt: *M.transandicum*; Mp: *M.panamense*; Mi: *M.inca*; Mg: *M.gallus*; Ma: *M.americanum*; Md: *M.digueti*; Ph: *P.hancocki*. **fig. S12.** Phylogenetic tree based on Bayesian inference approach generated under the GTR+I+G substitution model using concatenated dataset of the 16S rRNA-28S rRNA genes from six *Macrobrachium* species collected in Peruvian rivers of the Pacific. Bootstrap values and posterior probabilities ≥ 50% are shown. *P.hancocki* was used as outgroup. Abbreviations: Mt: *M.transandicum*; Mp: *M.panamense*; Mi: *M.inca*; Mg: *M.gallus*; Ma: *M.americanum*; Md: *M.digueti*; Ph: *P.hancocki*. https://binary.pensoft.net/file/1224230This dataset is made available under the Open Database License (http://opendatacommons.org/licenses/odbl/1.0/). The Open Database License (ODbL) is a license agreement intended to allow users to freely share, modify, and use this Dataset while maintaining this same freedom for others, provided that the original source and author(s) are credited.Eliana Zelada-Mázmela, Lorenzo E. Reyes-Flores, Luis De Stefano-Beltrán

Supplementary material 2 Additional information Data type 7z Explanation note **table S1.** Coordinates of collection sites of *Macrobrachium* species in rivers of the Peruvian coast. **table S2.***Macrobrachium* species identified in this study by morphological taxonomy 230 and molecular analyses. Sampling number (n), Peruvian rivers surveyed, and GenBank 231 accession numbers for each locus are shown. **table S3.** Key to species of *Macrobrachium* from Peru (Méndez 1981; Valencia and Campos 2007). **table S4.** Substitution model results based on JModel Test 2 analysis used for Bayesian inference approach. https://binary.pensoft.net/file/1224231This dataset is made available under the Open Database License (http://opendatacommons.org/licenses/odbl/1.0/). The Open Database License (ODbL) is a license agreement intended to allow users to freely share, modify, and use this Dataset while maintaining this same freedom for others, provided that the original source and author(s) are credited.Eliana Zelada-Mázmela, Lorenzo E. Reyes-Flores, Luis De Stefano-Beltrán
